# Donʼt be overconfident about the “cover” of a covered self-expandable metal stent in endoscopic ultrasound-guided hepaticogastrostomy

**DOI:** 10.1055/a-2505-9180

**Published:** 2025-01-16

**Authors:** Masaki Miyazawa, Masahiro Yanagi, Masaki Nishitani, Tomoyuki Hayashi, Shinya Yamada, Hajime Takatori, Taro Yamashita

**Affiliations:** 1 88335 Gastroenterology, Kanazawa University Hospital, Kanazawa, Japan

The usefulness of endoscopic ultrasound-guided hepaticogastrostomy (EUS-HGS) for biliary obstruction has been widely reported in cases where a transpapillary approach is unsuitable or impossible 1 2 . Covered self-expandable metal stents (CSEMSs) are often used for EUS-HGS, and the presence of the cover provides peace of mind that bile leakage will not occur, even if the CSEMS passes through the abdominal cavity 3 ; however, we report here a case in which, despite successful EUS-HGS using a CSEMS, biliary peritonitis occurred immediately afterward owing to a broken “cover” ( Video 1 ).

A case in which, despite successful endoscopic ultrasound-guided hepaticogastrostomy using a covered self-expandable metal stent for biliary obstruction due to unresectable distal biliary cancer, biliary peritonitis occurred immediately afterward owing to a broken “cover.”Video 1

A 65-year-old man diagnosed with unresectable distal biliary cancer developed obstructive jaundice and underwent transpapillary CSEMS placement ( Fig. 1 ); however, he developed cholangitis due to biliary hemorrhage and, at the time of reintervention, the tumor had invaded the duodenum, making the transpapillary approach impossible. Therefore, we performed biliary drainage by EUS-HGS. The intrahepatic bile duct B3 was punctured with a 19-gauge needle, and a 0.025-inch guidewire was inserted ( Fig. 2 **a** ). After the fistula had been dilated with a drill dilator ( Fig. 2 **b** ), a CSEMS (8 × 120 mm; Hanarostent Biliary Partial Cover Benefit; Boston Scientific, Massachusetts, USA) was quickly placed using the intrascope channel release technique ( Fig. 2 **c, d** ).

**Fig. 1:**
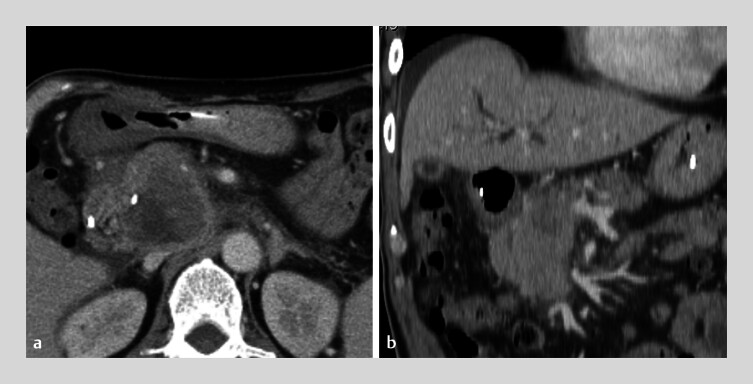
Computed tomography images showing obstructive jaundice due to distal biliary cancer.

**Fig. 2:**
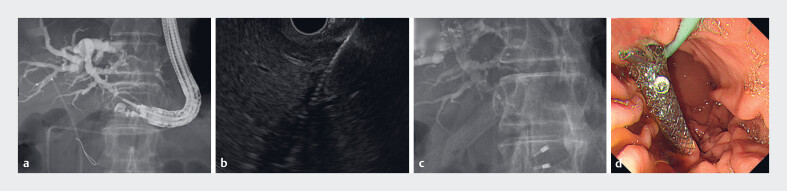
Images during biliary drainage using endoscopic ultrasound-guided hepaticogastrostomy showing: **a** a 0.025-inch guidewire inserted into the common bile duct after puncture of B3; **b** fistula dilation with a drill dilator; **c, d** a covered self-expandable metal stent, with a 5.9-cm thin delivery system and a 15-mm uncovered portion at the hepatic tip, placed from the B3 bile duct to the stomach.

All of the steps of the procedure were completed without any problems; however, the patient complained of fever and abdominal pain, and a computed tomography scan on the following day revealed ascites and free air in the abdominal cavity ( Fig. 3 **a** ). The bilirubin level in the ascites was high, so it was thought to be biliary peritonitis. Fistulography revealed contrast leakage from the CSEMS passing through the abdominal cavity ( Fig. 3 **b, c** ). Additional stenting was performed to cover the leak ( Fig. 3 **d** ), and the biliary peritonitis improved with peritoneal drainage and antibiotic treatment.

**Fig. 3:**
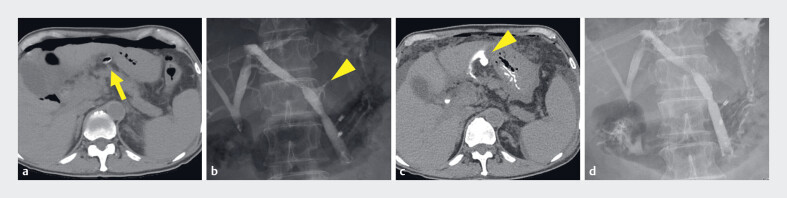
Images after the patient developed biliary peritonitis showing: **a** on computed tomography the following day, ascites and free air in the abdominal cavity, with the covered self-expandable metal stent (CSEMS) having passed through the abdominal cavity between the stomach and liver (arrow), although its position had not changed from the day of the ultrasound-guided hepaticogastrostomy (EUS-HGS); **b, c** on fistulography performed 2 days after the EUS-HGS, contrast leakage from the CSEMS passing through the abdominal cavity (arrowhead); **d** an additional stent placed to cover the leak.

This case taught us that we should not be overconfident about the “cover” of a CSEMS in EUS-HGS.

Endoscopy_UCTN_Code_CPL_1AL_2AD

## Notes

Endoscopy E-Videos https://eref.thieme.de/e-videos
*E-Videos* is an open access online section of the journal *Endoscopy* , reporting on interesting cases and new techniques in gastroenterological endoscopy. All papers include a high-quality video and are published with a Creative Commons CC-BY license. Endoscopy E-Videos qualify for HINARI discounts and waivers and eligibility is automatically checked during the submission process. We grant 100% waivers to articles whose corresponding authors are based in Group A countries and 50% waivers to those who are based in Group B countries as classified by Research4Life (see: https://www.research4life.org/access/eligibility/ ). This section has its own submission website at https://mc.manuscriptcentral.com/e-videos .
